# Targeting Myeloid-Derived Suppressor Cells via Dual-Antibody Fluorescent Nanodiamond Conjugate

**DOI:** 10.3390/nano14181509

**Published:** 2024-09-17

**Authors:** Colin D. Angell, Gabriella Lapurga, Steven H. Sun, Courtney Johnson, Himanshu Savardekar, Isaac V. Rampersaud, Charles Fletcher, David Albertson, Casey Ren, Lorena P. Suarez-Kelly, Arfaan A. Rampersaud, William E. Carson

**Affiliations:** 1 The Arthur G. James Comprehensive Cancer Center and Solove Research Institute, The Ohio State University, Columbus, OH 43210, USA; colin.angell@osumc.edu (C.D.A.); glapurg@rockets.utoledo.edu (G.L.); shsunsta@gmail.com (S.H.S.); courtney.johnson@wright.edu (C.J.); himanshu.savardekar@osumc.edu (H.S.); caseyren@stanford.edu (C.R.); 2 Department of Surgery, The Ohio State University, Columbus, OH 43210, USA; 3 Columbus NanoWorks, Inc., 1507 Chambers Road, Columbus, OH 43212, USA; isaacvcr@gmail.com (I.V.R.); charles@columbusnanoworks.com (C.F.); davenami@gmail.com (D.A.); arfaan@columbusnanoworks.com (A.A.R.); 4 Department of Surgery, University of Nevada, Las Vegas, NV 89102, USA; lsuarezkelly@gmail.com

**Keywords:** fluorescent nanodiamonds, antibody conjugation, MDSC

## Abstract

Fluorescent nanodiamonds (FNDs) are carbon-based nanomaterials that emit bright, photostable fluorescence and exhibit a modifiable surface chemistry. Myeloid-derived suppressor cells (MDSCs) are an immunosuppressive cell population known to expand in cancer patients and contribute to worse patient outcomes. To target MDSC, glycidol-coated FND were conjugated with antibodies against the murine MDSC markers, CD11b and GR1 (dual-Ab FND). In vitro, dual-Ab FND uptake by murine MDSC was significantly higher than IgG-coated FND (94.7% vs. 69.0%, *p* < 0.05). In vivo, intra-tumorally injected dual-Ab FND primarily localized to the tumor 2 and 24 h post-injection, as measured by in vivo fluorescence imaging and flow cytometry analysis of the spleen and tumor. Dual-Ab FND were preferentially taken up by intra-tumoral MDSC, representing 87.1% and 83.0% of FND+ cells in the tumor 2 and 24 h post-injection, respectively. Treatment of mice with anti-PD-L1 immunotherapy prior to intra-tumoral injection of dual-Ab FND did not significantly alter the uptake of FND by MDSC. These results demonstrate the ability of our novel dual-antibody conjugated FND to target MDSC and reveal a potential strategy for targeted delivery to other specific immune cell populations in future cancer research.

## 1. Introduction

Myeloid-derived suppressor cells (MDSCs) are immature myeloid cells with immunosuppressive functions that have been shown to expand in tumor-bearing hosts in response to tumor-derived factors [1,2]. The prevalence of MDSC is increased in patients with breast cancer, gastrointestinal cancer, glioblastoma, renal cell carcinoma, hepatocellular carcinoma (HCC), non-small cell lung carcinoma (NSCLC), prostate cancer, and melanoma [3,4,5,6,7,8,9,10]. Cytokines, chemokines, and metabolites produced by tumor cells lead to aberrant myelopoiesis, which results in the generation, expansion, and recruitment of MDSC to the tumor site. MDSC are recruited to the tumor by chemokines such as CXCL3, CXCL5, CXCL12, CCL2, and CCL5, where these cells mediate their immune-suppressive effects [11]. In humans, MDSC are characterized as CD33^+^, CD11b^+^, and HLA-DR^low/neg^ [1,12,13]. In mice, MDSC are characterized by GR1 and CD11b expression [14]. MDSC can suppress immune cell function utilizing a variety of mechanisms. This includes the generation of reactive oxygen species (ROSs) and nitric oxide (NO), the secretion of IL-10 and TGF-β, and the overexpression of amino acid-depleting enzymes arginase and indoleamine-pyrrole 2,3-dioxygenase (tryptophan), all of which can lead to the inhibition of T cell function [15]. Studies in murine models indicate that disruption of MDSC function can reverse immune tolerance to tumor antigens, stimulate anti-tumor immune responses, and improve the efficacy of immune-based therapies such as cancer vaccines and immune checkpoint inhibitors [2,16].

Fluorescent nitrogen vacancy (NV) center nanodiamonds (FNDs) are generated through electron irradiation and annealing of synthetic high-pressure, high-temperature diamonds [17]. NV centers emit bright, photostable fluorescence centered at ∼700 nm in the near-infrared (NIR) [18,19,20,21]. FND are chemically inert and display minimal cytotoxicity in vivo, as demonstrated previously by our group and others [20,22,23,24,25]. Additionally, FND do not show evidence of photobleaching after continuous excitation or fluorescence blinking and are brighter than organic dyes on a molar basis. They exhibit a longer lifetime, higher quantum yield, and infinite photostability compared to organic dyes, such as indocyanine green, and are at least comparable to quantum dots in physical properties [19]. These properties compare favorably to most commercial fluorophores and fluorescent proteins [19,25,26,27]. Thus, FND are being investigated as fluorescent probes for biomarkers and as theragnostic/therapeutic drug delivery agents. 

The biological applications of FND are enhanced by the high surface area-to-volume ratio of the nanoparticles themselves and their robust surface chemistry, which allows conjugation to a variety of molecules [26,27]. To improve biocompatibility, nanodiamonds can be coated with non-toxic molecules, that is, silica or polymers, such as polyethylene glycol [PEG] or glycidol [23,28,29,30,31,32]. Glycidol is a three-carbon epoxy alcohol that creates a dense hydrophilic coating around FND, which minimizes non-specific interactions [23,29,30,33], mitigates the tendency to aggregate in physiological conditions [29,33], and provides functional groups for conjugation with DNA, proteins, or therapeutic drugs [30,31]. 

The tumor microenvironment is characterized by complex interactions between various immune cell populations and tumor cells. We propose that FND can be utilized in cancer immunotherapy to target and modulate immune cell activity, especially in those with endocytic capacity, such as MDSC [34,35]. Our group has created a novel, modified FND targeted to murine MDSC by conjugating antibodies recognizing murine CD11b and GR1. We hypothesized that the binding of the antigen recognition portion of these antibodies to surface-expressed CD11b and GR1 would result in the localization of FND to MDSC and greater specificity of uptake. The present manuscript details the creation of this reagent and its utility in experimental murine models.

## 2. Materials and Methods

### 2.1. Chemicals and Reagents

Chemicals and solvents were purchased from GFS Chemicals (Columbus, OH, USA) and Sigma-Aldrich (St. Louis, MO, USA). Anti-GR1, anti-CD11b, and human IgG were purchased from Southern Biotech (Birmingham, AL, USA). The polyethylene glycol (PEG) linker, azido-PEG_12_-NHS ester, was purchased from Broadpharm (San Diego, CA, USA). NHS-biotin was purchased from Quanta Biodesign (Powell, OH, USA), epichlorohydrin was purchased from Alfa Aesar (Haverhill, MA, USA), and N,N’-disuccinimidyl carbonate (DSC) was purchased from Novabiochem (now Sigma-Aldrich, St. Louis, MO, USA). Glycidol was a gift from Dixie Chemical Co. (Pasadena, TX, USA) and was redistilled under reduced pressure at 31 °C prior to use.

### 2.2. GPE Synthesis

Glycidyl propargyl ether (GPE) was synthesized by reacting propargyl alcohol with epichlorohydrin in the presence of a strong base [36]. The crude GPE was purified by vacuum distillation, with pure GPE coming off between 25 °C and 30 °C. The presence of the triple bond and epoxy group on the GPE was confirmed by Fourier-transform infrared spectroscopy (FT-IR) [37]. Mass spectroscopy was performed on the synthesized GPE using a Waters SYNAPT XS (Waters Corp, Milford, MA, USA). A solution of 10 μg/mL GPE in acetonitrile was prepared and measured using direct injection at 10 μL/min in positive ion configuration. Mass spectroscopy was also performed on commercial GPE (part number G0445, TCI America, Portland, OR, USA) under the same conditions (Figure S1).

### 2.3. FND Synthesis

FND were fabricated, as previously described [17]. Briefly, 100 nm synthetic high-pressure, high-temperature diamonds were bombarded with electrons, delivered at a dose of 2 × 10^18^ e/cm^2^, and then annealed at 800 °C for two hours in argon gas [17]. FND were cleaned by refluxing for 72 h at 70 °C in a 9:1 mixture of concentrated sulfuric acid and concentrated nitric acid. FND were diluted with water and centrifuged in a Beckman TJ-6 bench-top centrifuge (Beckman, Brea, CA, USA) equipped with a TH-4 horizontal rotor (2700 RPM for 30 min). FND were repeatedly resuspended with pure water and recentrifuged until the pH of the supernatant was neutral. FND were analyzed for hydrodynamic size on a Brookhaven 90 Plus particle size analyzer (Brookhaven Instruments, Nashua, NH, USA) with BIC particle sizing software ver 4.08 using a 660 nm laser and a 90° incident angle. Fluorescence spectra were collected on a home-built confocal microscope.

### 2.4. Amination of Glycidol-Coated FND

FND were coated with glycidol to introduce reactive alcohol groups for subsequent conjugation steps [38]. Approximately 1 mg of glycidol-coated FND was suspended in a 1:1 mixture of dimethylacetamide and tetrahydrofuran (DMAC/THF), centrifuged, and then resuspended in a mixture containing 0.1 mL of DMAC and 0.9 mL of dimethyl formamide. The nanodiamonds were activated with 0.2 mmol of DSC. After the final rinse in DMAC/THF solvent, the nanodiamonds were quickly resuspended in 50 mM HEPES, pH 7.4, containing 0.05% Tween 20 (HEPEST), and then 0.18 mmol of 4,7,10-Trioxa-1,13-tridecanediamine was added. After shaking for 4 h at room temperature, the aminated diamonds were rinsed and resuspended in DMAC/THF solvent.

### 2.5. Creation of Aminated-Propargyl FND

The propargyl group of GPE was introduced on the diamond surface through the reaction of the epoxy group of GPE with the aminated FND. A limited reactivity was noted between epoxides and aminated FND in 80 mM carbonate-bicarbonate buffer, pH 8.5, and this buffer was used to create FND having both amine and propargyl groups on the diamond surface, aminated-propargyl FND. Aminated FND were resuspended in 1 mL of an 80 mM carbonate-bicarbonate buffer, pH 8.5, and approximately 0.40 mmole of GPE was added. The solution was allowed to react overnight with vigorous shaking. The samples were centrifuged, and the nanodiamonds were washed and resuspended with water.

### 2.6. Amine Assay

Succinimidyl 3-(2-pyridyldithio) propionate (SPDP) was used to detect the remaining amines on the surface of FND. Briefly, aminated FND were suspended in 1 mL phosphate-buffered saline containing 1 mM EDTA (PBS-EDTA), and 25 µL of a 20 mM SPDP (in DMAC) was added. Following a 2 h incubation at room temperature, the mixture was centrifuged, the FND pellet rinsed, and then resuspended in PBS-EDTA. Ten microliters of 15 mg/mL dithiothreitol (DTT) were added, and samples were mixed on a Vortex Genie 2 shaker (Scientific Industries, INC, Bohemia, NY, USA) for 15 min. The DTT cleaved pyridine-2-thione from the SPDP-nanodiamond adduct, which was separated from the nanodiamonds by centrifugation at 20,800 RCF. Triplicate 200 µL aliquots of supernatant were put in a 96-well plate, and the absorbance at 350 nm was read using a Tecan GENios plus microplate reader (Tecan US, Morrisville, NC, USA) with Magellan 7.2 software. A standard curve was created by making serial dilutions of 20 mM SPDP solution in PBS-EDTA, which were then reduced with DTT, and the absorbance was read at 350 nm.

### 2.7. Fourier-Transform Infrared Spectroscopy (FT-IR)

All FT-IR spectra were obtained on an Agilent 4500 FT-IR (Agilent, Santa Clara, CA, USA) with a diamond ATR. Briefly, the diamond ATR chip was cleaned using 70% ethanol, and a background FT-IR spectrum was obtained. The nanodiamond sample was suspended in dH_2_O at 10 mg/mL. Two microliters of the suspension were pipetted onto the ATR chip and dried under a gentle vacuum, and then 2 µL of the suspension was added again and dried under a gentle vacuum. The FT-IR spectrum for the sample was then gathered.

### 2.8. Creation of PEGylated GR1 Antibody

Approximately 10 µg of anti-GR1 IgG (0.1 nmol) was diluted with 400 µL of HEPEST and then reacted with a 10-fold molar excess of azido-PEG_12_-NHS ester at room temperature for 2 h. The reaction was transferred to a 10,000 MWCO spin filter (Spin-X UF 500, Corning, Corning, NY, USA), and the unconjugated PEG linker was removed by washing the filter with HEPEST. The azido-PEG_12_-anti-GR1 conjugate was recovered from the spin filter by resuspension in 200 µL of HEPEST buffer. The concentration of the conjugate was estimated at 50 µg/mL.

### 2.9. Bioconjugation Reactions

Conjugations to the aminated groups on the bi-functionalized FND were completed before performing the Click reactions. Approximately 0.2 mmol of DSC was incubated with 1 mg of aminated FND in 1 mL of DMAC/THF at room temperature for 4 h. The FND were rinsed thrice with DMAC/THF before quickly resuspending in 1 mL HEPEST buffer and adding 2 µg of anti-CD11b antibodies. The mixture was incubated for 2 h at room temperature, after which the resulting nanodiamond immunoconjugate was centrifuged, resuspended in 500 µL of HEPEST, and then divided into five 100 µL aliquots.

In a related set of reactions, NHS-PEG_12_-biotin was conjugated, instead of anti-CD11b antibodies, to the aminated-propargyl FND. In these experiments, DSC activation of the FND was not performed. Briefly, 1 mg of aminated-propargyl FND was mixed with 10 µL of 1 mg/mL NHS-PEG_12_-biotin and allowed to react for 2 h. These biotinylated FND were rinsed thrice, resuspended in 500 µL HEPEST, and divided into five 100 µL aliquots.

Click reactions were performed on both CD11b-conjugated FND and biotinylated FND. Approximately 2 mg of PEGylated GR1 antibody was added to each tube containing 100 µL of FND and allowed to react for 5 min. Approximately 50 µL of 20 mg/mL (46 mM) tris-hydroxypropyl-triazolylmethylamine (THPTA) was mixed with 25 µL of 5 mg/mL (31.3 mM) copper sulfate and allowed to react for 5 min. Fifteen microliters of the THPTA/CuSO_4_ mixture were added to each tube of FND solution and quickly followed with 12 mL of 5 mg/mL (25.2 mM) sodium L-ascorbate. The resulting mixture was incubated for 24 h at 4 °C with vigorous shaking, centrifuged, and resuspended in HEPEST buffer containing 1 mM ethylenediaminetetraacetic acid (EDTA). The EDTA was then removed by rinsing twice in HEPEST buffer and resuspending in 200 µL HEPEST buffer.

In one set of studies, only anti-GR1 antibodies were conjugated to the aminated-propargyl FND. This immunoconjugate was created by first reacting 1 mg of aminated-propargyl FND with 10 µL of 1 mg/mL m-dPEG2-NHS ester for 2 h to block available surface amines. The anti-GR1 antibody was attached through the Click reactions, as described above.

In a related set of studies, approximately 1 mg of glycidol-coated FND was suspended in a 1:1 mixture of dimethylacetamide and tetrahydrofuran (DMAC/THF), centrifuged, and then resuspended in a mixture containing 0.1 mL of DMAC and 0.9 mL of dimethyl formamide. The nanodiamonds were activated with 0.2 mmol of DSC, as described previously. After the final rinse in DMAC/THF solvent, the nanodiamonds were quickly rinsed in HEPEST buffer, resuspended in 1 mL HEPEST, and 20 ug of human IgG (Southern Biotech, Birmingham, AL, USA) was added. After shaking for 4 h at room temperature, the hu-IgG-coated FND (IgG FND) were rinsed three times and resuspended in 1 mL HEPEST buffer.

### 2.10. Modified ELISA

A 10 mg/mL solution of streptavidin was prepared in 200 mM carbonate buffer, pH 9.4, and used to coat the wells of a 96-well polystyrene plate (Nunc, Thermo Fisher Scientific, Waltham, MA, USA) overnight at 4 °C. The wells were washed with HEPEST and blocked for 2 h with 300 µL of 20 mg/mL bovine serum albumin in the same buffer at room temperature.

FND were diluted to 100 µg/mL in rinse buffer containing 10 mg/mL BSA, and 100 µL of each glycidol-coated FND, anti-GR1 FND, biotinylated FND, and anti-GR1/biotinylated FND were added to each well. The plate was allowed to incubate for 2 h at room temperature with gentle shaking. Each well was then gently washed thrice with rinse buffer and incubated with 100 µL of goat anti-rat–HRP (diluted 1000× in HEPEST). Due to the close species-relatedness, the goat anti-rat IgG will cross-react with the mouse IgG. The plate was incubated for 2 h at room temperature with gentle shaking, and then each well was gently rinsed three times with rinse buffer. The wells were developed with 100 µL of a 3,3′,5,5′-tetramethylbenzidine (TMB) solution and were quenched with 100 µL of 1 M H_2_SO_4_. The absorbance in each well was read at 450 nm in a Tecan Genios Plus plate reader (Tecan US, Morrisville, NC, USA).

### 2.11. Kinetic HRP Procedure

Following bioconjugation of anti-CD11b antibody, but before conjugation with anti-GR1 antibody, 20 µg of FND (CD11b FND) was set aside. Twenty micrograms of glycidol-coated FND (gFND), CD11b FND, and the final anti-CD11b/anti-GR1 antibody dual conjugate (dual-Ab FND) were resuspended in 500 µL of 1 µg/mL of goat anti-rat IgG HRP conjugate (#3030-05, Southern Biotech, Birmingham, AL, USA) in HEPEST buffer (50 mM HEPES, 0.05% Tween 20). FND were incubated for 2 h at room temperature with shaking. The nanodiamonds were separated by centrifugation at 14,000 RCF for 7 min and resuspended in 500 µL HEPEST. This was repeated three additional times, resuspending in 100 µL HEPEST on the final rinse. A 1 mL quartz cuvette was loaded with 900 µL dH_2_O and 100 µL of TMB substrate mixture (peroxidase substrate #34021, Thermo-Scientific, Waltham, MA, USA). Then, 5 µL of the nanodiamond suspension was added, and the absorbance at 650 nm was measured using a Shimadzu UV-2101PC spectrophotometer (Shimadzu, Kyoto, Japan) and recorded for 120 s. This process was repeated three times for each sample. The slope of the absorbance curve was used to determine the relative HRP activity of each sample.

### 2.12. Cell Lines

The murine MDSC-like cell line MSC2 was provided by Gregoire Mignot (University of Burgundy, Dijon, France). The murine breast triple-negative breast cancer (TNBC) cell line EMT6 was obtained from the American Type Culture Collection (Manassas, VA, USA). Both cell lines were maintained in RPMI 1640 media supplemented with 10% FBS and 1% antibiotic-antimycotic (Life Technologies Inc., Rockville, MD, USA).

### 2.13. Isolation of MDSC from Tumor-Bearing Mice

Wild-type 4–6-week-old BALB/c mice (The Jackson Laboratory, Bar Harbor, ME, USA) were injected subcutaneously in the right dorsal flank with 1 × 10^6^ EMT6 breast cancer cells. Once tumors reached ~500 mm^3^ in size, the mice were euthanized. The spleens were harvested aseptically and processed into single-cell suspensions. Cell pellets were treated with RBC lysis buffer (BioLegend, San Diego, CA, USA) for 3 min at 24 °C. Following processing, CD11b^+^/GR1^+^ MDSC were isolated via magnetic negative selection using the Mouse MDSC (CD11b GR1) Isolation Kit (Stem Cell, Vancouver, BC, Canada), with an achievement of >90% purity as measured by flow cytometry.

### 2.14. In Vitro FND Treatment Assays

MSC2 cells were added to 6-well plates at a concentration of 1 × 10^6^ cells/well in complete RPMI 1640 media and cultured for 24 h with 25 µg each of uncoated FND (uFND), IgG FND, and dual-Ab FND. Purified splenic MDSC from EMT6 tumor-bearing mice (as described above) were added to a 24-well plate at a concentration of 1 × 10^6^ cells/well in complete RPMI 1640 media and cultured for 24 h with 25 µg each of uncoated FND (uFND), glycidol FND (gFND), IgG FND, CD11b FND, and dual-Ab FND. Cells were then collected for flow cytometry analysis.

### 2.15. In Vivo Localization of FND in a Murine Breast Cancer Model

Wild-type 4–6-week-old BALB/c mice were injected subcutaneously in the right dorsal flank with 1 × 10^6^ EMT6 breast cancer cells. Once tumors were approximately 1 cm in diameter, tumors were injected with 100 µL HEPEST buffer (no FND) or 100 µL 1 µg/µL uFND, IgG FND, or dual-Ab FND. One mouse from each group was euthanized 2 and 24 h after the intra-tumoral injection and imaged using the Maestro spectral imaging system (Capiler Life Sciences, Hopkinton, MA, USA) with Maestro 2.2 software to visualize and track the nanodiamonds. Spleens and tumors were aseptically harvested 2 and 24 h following intra-tumoral injection and processed into single-cell suspensions for flow cytometry analysis. These studies were performed in strict accordance with the recommendations in the Guide for the Care and Use of Laboratory Animals of the National Institutes of Health and were conducted under a protocol approved by Ohio State University’s Institutional Animal Care and Use Committee.

### 2.16. In Vivo FND Uptake by MDSC during PD-L1 Blockade

Six EMT6 tumor-bearing BALB/c mice were treated with anti-PD-L1 antibody via intra-peritoneal injections three times per week (Mondays/Wednesdays/Fridays) at a dose of 100 µg of antibody per injection. Six untreated tumor-bearing mice served as controls. Twenty-four hours after the fourth dose, mice were given intra-tumoral injections of 100 µL of 1 µg/µL uFND, IgG FND, or dual-Ab FND. Animals were sacrificed 24 h after the FND injection and then imaged using the Maestro spectral imaging system. After imaging, tumors and spleens were aseptically harvested, processed into single-cell suspensions, and analyzed via flow cytometry.

### 2.17. Flow Cytometry

Cells were stained with fluorochrome-labeled antibodies listed in Table S1. Flow cytometry was performed on an LSRFortessa flow cytometer (BD Biosciences, San Jose, CA, USA). Cellular FND uptake was measured using the PE-Cyanin 5.1 channel. 

### 2.18. Statistical Analysis

Analyses were performed using a repeated measures ANOVA and an ordinary one-way ANOVA for paired and unpaired tests, respectively. Tukey’s post hoc test was used to adjust for multiple comparisons. Statistical analyses were conducted using GraphPad Prism version 10.0.2 for Windows (GraphPad Software, Boston, MA, USA).

## 3. Results

### 3.1. Synthesis of FND Labeled with Two Distinct Antibodies

**Figure 1:**
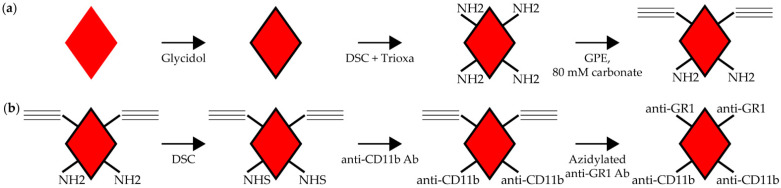
Synthesis of FND labeled with two antibodies. Shown is the schema for creating aminated FND with propargyl groups and the steps involved with creating dual-antibody-conjugated FND. (**a**) Glycidol-coated FND were reacted with N, N′-disuccinimidyl carbonate (DSC) and 4,7,10-Trioxa-1,13-tridecanediamine (Trioxa) to create FND with aminated groups on the surface. Limited epoxidation of the amines with glycidyl propargyl ether (GPE) in carbonate buffer introduced propargyl groups. (**b**) Amine groups on the FND were reacted with DSC to create NHS esters, then mixed with anti-CD11b antibodies to create the first immunoconjugate. Azido-PEG_12_-anti-GR1 antibodies were then reacted with the propargyl groups in a copper-catalyzed “Click” reaction to create the dual-Ab FND.

Figure 1 shows the overall synthetic pathways used to create multiple functional groups on the FND. Glycidol-coated FNDs containing both amine and propargyl groups were reacted with DSC to create NHS ester groups. These reactive groups were used to conjugate the anti-CD11b antibody to the diamond surface. The anti-GR1 antibody was then modified with an NHS-PEG_11_-azide and reacted with the propargyl groups on the diamond surface in a copper-catalyzed “Click” reaction. Once the reactions were complete, the modified FND were evaluated for the presence of the antibodies on the diamond surface.

Several approaches were evaluated to create different functional groups on nanodiamond particles. The most successful tactic was first to perform the epoxidation reaction between GPE and aminated FND in the presence of 80 mM carbonate buffer, pH 8.5. The carbonate buffer mediated the epoxidation reactions on the FND while also leaving free amine groups on the FND surface. To demonstrate this, we performed epoxidation reactions at increasing carbonate concentrations and measured the remaining amines using SPDP. The SPDP converts the amino groups to 2-pyridyl disulfide [39,40]. Reduction with DTT breaks the disulfide bond, releasing pyridine-2-thione. Released pyridine-2-thione can be measured by absorption at 350 nm, which is proportional to the number of amines remaining on the FND surface following the epoxidation reaction with GPE. Figure S2 shows the percent of remaining amines on the FND following epoxidation reactions at different carbonate concentrations. There was an inverse correlation between carbonate concentrations and the percentage of remaining amines. As the molarity of the buffer increased from 40 mM to 160 mM carbonate buffer, there was a notable decrease in the remaining amines. This result suggested that we could control the extent of epoxidation on the FND by adjusting the molarity of the carbonate buffer in the reaction. Based on the assay for amine groups, approximately 10% of the amine groups remained on the FND following reaction with GPE in 80 mM carbonate-bicarbonate buffer, pH 8.5. The free amines that remained on the FND could then be converted to reactive N-hydroxysuccinimide (NHS) esters through DSC activation.

An additional approach to demonstrate the successful conjugation of different functional groups on the FND is demonstrated in Figure S3. The glycidol-coated FNDs (orange line) have a broad peak at 3400 cm^−1^ from the –OH groups as well as two peaks at 2870 and 2920 cm^−1^ from the CH_2_ stretching in the polymerized glycidol [37]. These peaks remain following amination (blue line) and reaction with GPE (black line). The aminated FND (blue line) have a larger peak at 1650 than the glycidol-coated FND, indicating the NH_2_ bending mode. When the amine diamonds are reacted with GPE (black line), a small peak at 3270 appears, indicating the alkyne C-H stretching mode of the propargyl group. The peak at 1650 cm^−1^ is again larger for the propargyl, aminated FNDs (black line) than the same peak of the glycidol-coated FND (orange line), indicating there are amine groups that remain unreacted. Overall, these results indicate the presence of amine and propargyl groups on the FND surface following the epoxidation reaction with GPE.

### 3.2. ELISA of FND Dual Conjugation

**Figure 2:**
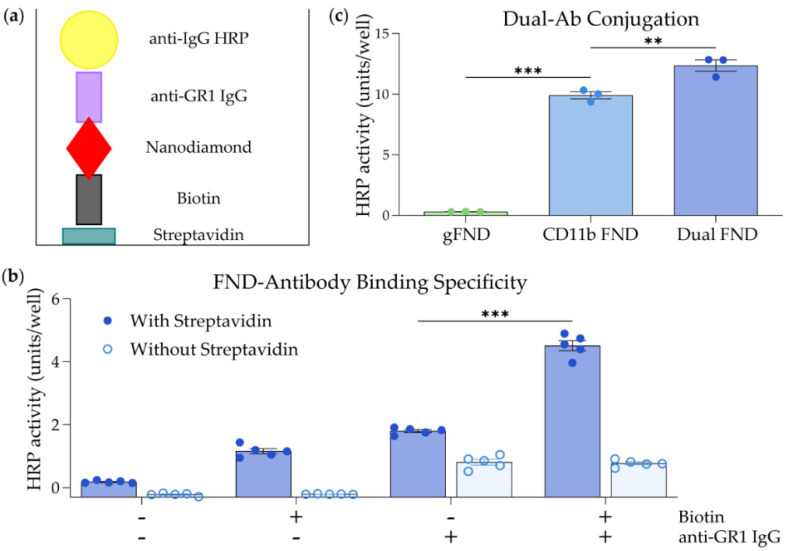
ELISA-based analysis of FND dual conjugation. (**a**) Schematic of the ELISA-based assay used to detect the combination of biotin and anti-GR1 IgG on the FND. (**b**) Biotinylated anti-GR1 FND was diluted to 100 µg/mL, added to streptavidin-coated wells, and reacted for 2 h at room temperature. Controls included glycidol-coated FND (Biotin- and anti-GR1 IgG-), biotinylated FND (Biotin+, anti-GR1 IgG-), and anti-GR1 FND (Biotin- and anti-GR1 IgG+). (**c**) Enzymatic activity of goat anti-rat-HRP was used to detect the presence of anti-CD11b and anti-GR1 Ab on glycidol-coated FND (gFND), anti-CD11b-conjugated FND (CD11b FND), and anti-CD11b/anti-GR1-conjugated FND (Dual FND). Note: Due to the close species-relatedness, the goat anti-rat IgG will cross-react with the mouse IgG. Bars indicate mean ± SEM. Statistical significance was analyzed by an ordinary one-way ANOVA followed by Tukey’s post hoc test. ** *p* < 0.01 and *** *p* < 0.001.

An ELISA-based assay was used to confirm the dual conjugation of antibodies to the FND surface. In the first assay, NHS-PEG_12_-biotin was conjugated instead of anti-CD11b antibodies to the aminated-propargyl FND. Successful conjugation of biotin to the FND results in anchoring of the FND to streptavidin-coated wells, while successful conjugation of anti-GR1 Ab allows for binding of anti-IgG goat anti-rat-HRP antibody (Figure 2a). The biotinylated anti-GR1 FND was diluted to 100 µg/mL, added to streptavidin-coated wells, and reacted for 2 h at room temperature. Controls included glycidol-coated FND, anti-GR1-only FND, and biotinylated-only FND. The enzymatic activity of goat anti-rat-HRP was used to detect the presence of the anti-GR1 Ab on the plated FND. The highest levels of HRP activity were generated for the FND, which displayed both anti-GR1 Ab and biotin (Figure 2b). The other constructs produced lower levels of activity that were similar to background levels seen when streptavidin was added to empty wells. To further confirm successful dual conjugation, goat anti-rat-HRP was used to detect the presence of antibodies after each conjugation step. Glycidol-coated FND (gFND), anti-CD11b-conjugated FND (CD11b FND), and dual conjugated anti-CD11b/anti-GR1 FND (Dual FND) were incubated with goat anti-rat-HRP for 2 h, and HRP enzymatic activity was measured. There was a significant increase in HRP activity following both CD11b conjugation (gFND vs. CD11b FND, *p* < 0.001) and GR1 conjugation (CD11b FND vs. Dual FND, *p* < 0.01) (Figure 2c). These results demonstrate the successful dual conjugation of anti-CD11b and anti-GR1 antibodies to the surface of FND.

### 3.3. In Vitro FND Uptake Assays

**Figure 3:**
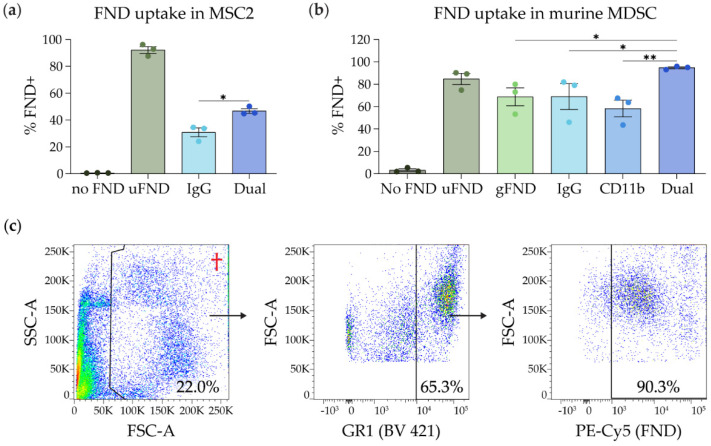
In vitro FND treatment assays with MSC2 and purified murine MDSC with an example gating strategy. (**a**) 1 × 10^6^ MSC2 cells were cultured for 24 h with 25 µg of the following FND: unmodified FND (uFND), IgG FND, or dual CD11b/GR1 antibody FND (Dual FND) (n = 3). Uptake of FND by MSC2 was evaluated via flow cytometry on the PE-Cyanin 5.1 channel. Statistical significance was analyzed by an ordinary one-way ANOVA followed by Tukey’s post hoc test. (**b**) 1 × 10^6^ purified splenic MDSC from three tumor-bearing mice were cultured for 24 h with 25 µg of the following FND: uFND, gFND, IgG FND, CD11b FND, or dual-Ab FND (n = 3). Uptake of FND by GR1^high^ MDSC was evaluated via flow cytometry. (**c**) Representative flow cytometry gating of murine MDSC treated with FND. † Note: Debris and free FNDs in solution have low FSC-A and were excluded with the first gate (FSC-A vs. SSC-A). FNDs increase SSC-A, resulting in cells positive for FND appearing at the top of the first gate (FSC-A vs. SSC-A). Statistical significance was analyzed by a repeated measures ANOVA followed by Tukey’s post hoc test. Bars indicate mean ± SEM. * *p* < 0.05 and ** *p* < 0.01. SSC-A, side scatter area; FSC-A, forward scatter area.

To quantify the uptake of FND by MDSC, FND were cultured with MSC2 cells and murine MDSC. MSC2 cells are an immortalized murine cell line of CD11b^+^/GR1^+^ immunosuppressive myeloid cells used to model MDSC [41,42,43]. MSC2 cells were cultured for 24 h with 25 µg of the following FND: uFND, IgG FND, or dual-Ab (CD11b/GR1) FND. The uptake of FND was evaluated via flow cytometry on the PE-Cyanin 5.1 channel at the 667 nm emission wavelength. The greatest uptake in MSC2 cells was seen with uFND at an average rate of 92.2%, compared to 30.8% with IgG FND and 46.8% with dual-Ab FND (Figure 3a). There was a significant increase in uptake of dual-Ab FND compared to IgG FND (*p* < 0.05). Purified splenic MDSC from three EMT6 tumor-bearing mice were cultured for 24 h with 25 µg of the following FND: uFND, gFND, IgG FND, CD11b FND, or dual-Ab (CD11b/GR1) FND. Following the 24 h incubation, FND uptake by GR1^high^ MDSC was evaluated via flow cytometry. MDSC cultured with dual-Ab FND averaged the highest uptake at 96.2%. Uptake by uFND, gFND, IgG FND, and CD11b FND was 84.7%, 68.7%, 69.0%, and 58.3%, respectively. Dual-Ab FND uptake was significantly higher than gFND (*p* < 0.05), IgG FND (*p* < 0.05), and CD11b FND (*p* < 0.01) (Figure 3b). Representative flow cytometry gating is shown in Figure 3c.

### 3.4. In Vivo Localization of FND in Tumor-Bearing Mice

**Figure 4:**
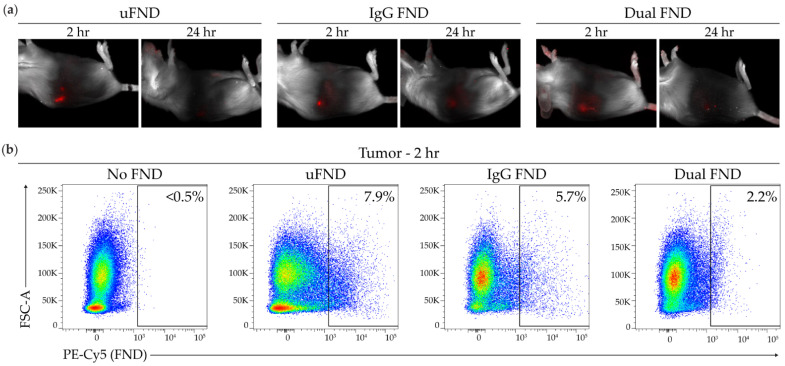

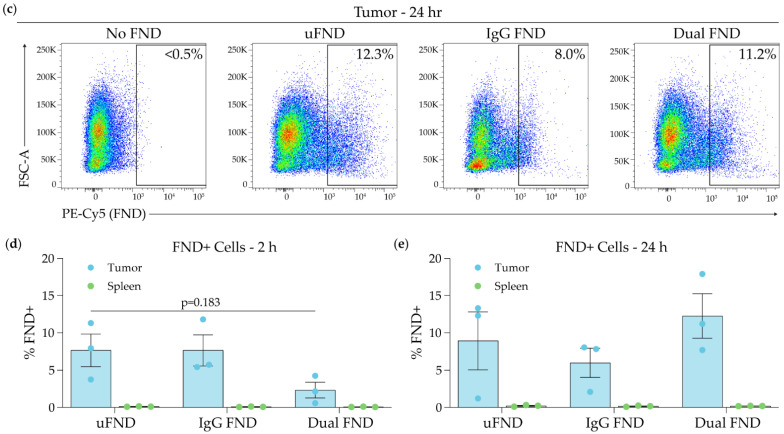
Localization of FND in tumor-bearing mice. 4–6-week-old female BALB/c mice were injected subcutaneously in the dorsal flank with 1 × 10^6^ EMT6 breast cancer cells. An intra-tumoral injection of 100 µL of HEPEST buffer (No FND) or 100 µL of 1 µg/µL of fluorescent nanodiamonds (uFND, IgG FND, or dual-Ab FND) was administered once tumors were approximately 1 cm in diameter. (**a**) Fluorescence imaging of FND using the Maestro spectral imaging software 2 and 24 h following injections. (**b**–**e**) Tumors and spleens were harvested at 2 and 24 h following injection, processed into single-cell suspension, and analyzed via flow cytometry for FND uptake. Representative flow cytometry plots demonstrating the percentage of FND+ cells in the tumor (**b**) 2 and (**c**) 24 h following the injection. Summary of the percentage of FND+ cells (**d**) 2 and (**e**) 24 h following injection. Bars indicate mean ± SEM. Statistical significance was analyzed by an ordinary one-way ANOVA followed by Tukey’s post hoc test. FSC-A, forward scatter area.

To visualize the localization of FND in vivo, mice were imaged using the Maestro spectral imaging software following intra-tumoral injection of FND. EMT6 tumors were subcutaneously implanted in the dorsal flank of 4–6-week-old BALB/c mice. Once tumors were approximately 1 cm in diameter, 100 µL of uFND, IgG FND, or dual-Ab FND at a concentration of 1 µg/µL was injected into the tumor, and FND localization was captured 2 and 24 h following injection. All three groups had a strong signal 2 h following injection (Figure 4a). At 24 h following injection, mice injected with IgG FND and dual-Ab FND had reduced fluorescence, while the mouse injected with uFND had no detectable fluorescence. To determine cellular uptake of FND following intra-tumoral injection, FND+ cells in the tumor and spleen were quantified via flow cytometry. Tumors and spleens were harvested from EMT6-bearing mice 2 and 24 h following intra-tumoral injection of either 100 µL buffer containing no FND or 100 µL of uFND, IgG FND, or dual-Ab FND at a concentration of 1 µg/µL. Tumors and spleens were processed into single-cell suspensions and analyzed via flow cytometry for FND uptake, as shown in Figure S4. At 2 h, 7.9% and 5.7% of all tumor and immune cells in representative tumors were FND+ following treatment with uFND and IgG FND, respectively. Approximately 2.2% of all tumor and immune cells were FND+ in a representative tumor treated with dual-Ab FND (uFND vs. dual-Ab FND, *p* = 0.183) (Figure 4b,d). FND positivity of all tumor and immune cells in the tumor increased slightly at 24 h (Figure 4c,e). Representative tumors injected with uFND, IgG FND, and dual-Ab FND had 12.3%, 8.0%, and 11.2% FND positivity in tumor/immune cells, respectively (Figure 4c). FND uptake in the spleen was undetectable (<0.5% FND+) at both 2 and 24 h in all three groups (Figure 4d,e and Figure S5). These results indicate that intra-tumorally injected FND localizes to the tumor and remains in the tumor for up to 24 h post-injection.

### 3.5. Specificity of Dual-Antibody Conjugated to MDSC in the Tumor Microenvironment

**Figure 5:**
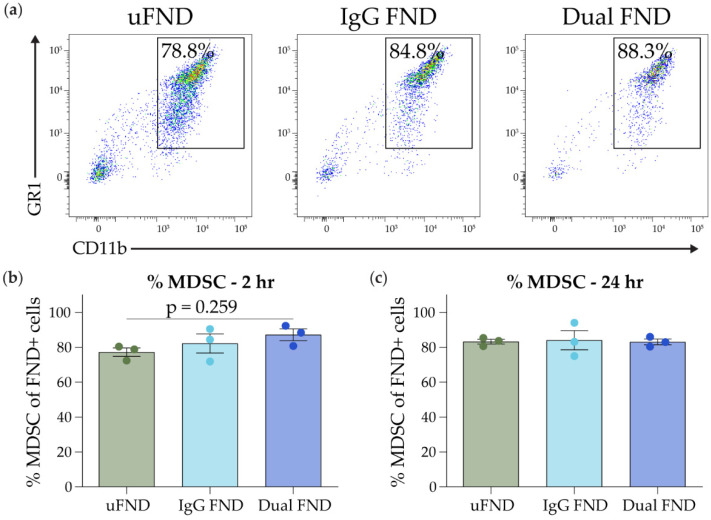
Specificity of dual-antibody conjugated FND to MDSC in the tumor microenvironment. Mice bearing EMT6 tumors were treated as described in Figure 4. (**a**) Representative flow plots demonstrating the percentage of CD11b^+^/GR1^+^ cells among FND+ cells in the tumor 2 h following an intra-tumoral injection with 100 µL of 1 µg/µL uFND, IgG FND, and dual-Ab FND. (**b**,**c**) Summary of the percentage of CD11b^+^/GR1^+^ cells among FND+ cells in the tumor (**b**) 2 and (**c**) 24 h following injection. Bars indicate mean ± SEM. Statistical significance was analyzed by an ordinary one-way ANOVA followed by Tukey’s post hoc test. FSC-A, forward scatter area.

To evaluate the ability of dual-Ab FND to target MDSC in the tumor microenvironment preferentially, the percentage of CD11b^+^/GR1^+^ MDSC among all FND+ cells in the tumor was analyzed via flow cytometry. As described above, EMT6 tumors injected with uFND, IgG FND, or dual-Ab FND were harvested 2 and 24 h following injection, processed into single-cell suspension, and the percentage of MDSC among FND+ cells was analyzed by flow cytometry (Figure S6). All three types of FND demonstrated high specificity to MDSC, with the majority (75–90%) of FND+ cells in the tumor 2 and 24 h following injection being CD11b^+^/GR1^+^ MDSC (Figure 5b,c). At 2 h following injection, there was a trend toward an increase in the percentage of MDSC among FND+ cells in tumors treated with dual-Ab FND compared to uFND or IgG FND (uFND: 77.2% ± 2.4; IgG FND: 82.2% ± 5.5; dual-Ab FND: 87.1% ± 3.4; uFND vs. dual-Ab FND, *p* = 0.259) (Figure 5a,b). At 24 h following injection, all three types of FND had approximately 83% of their uptake by MDSC (Figure 5c and Figure S7). These results indicate that MDSC are the primary cell type that take up FND in the tumor and that dual-Ab conjugation could improve targeting specificity at 2 h following injection.

### 3.6. FND Uptake by MDSC during the Course of PD-L1 Blockade

**Figure 6:**
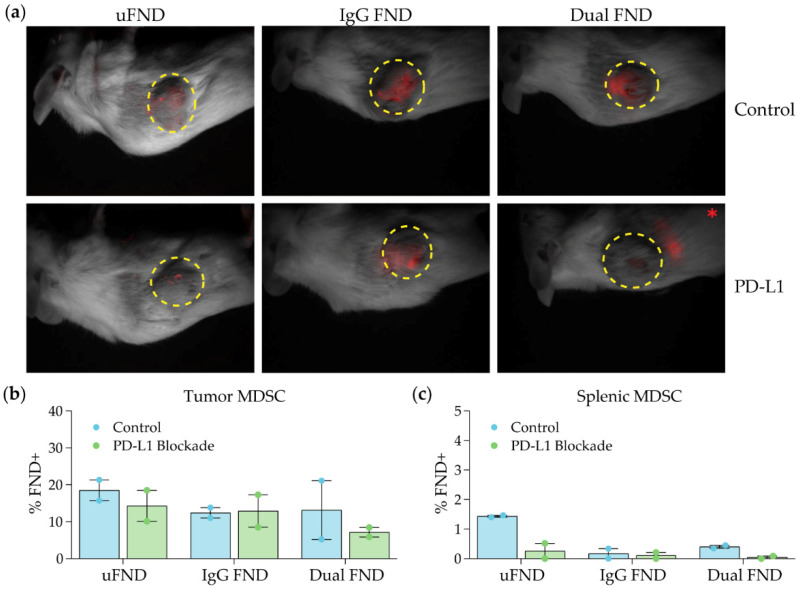
FND uptake by MDSC during PD-L1 blockade. Six EMT6 tumor-bearing female BALB/c mice were treated with anti-PD-L1 antibody via intra-peritoneal injections three times per week (Mondays/Wednesdays/Fridays) at a dose of 100 µg of antibody per injection. Six untreated tumor-bearing mice served as controls. Twenty-four hours after the fourth dose, mice were given an intra-tumoral injection of 100 µL of 1 µg/µL uFND, IgG FND, or dual-Ab FND. (**a**) Animals were sacrificed 24 h after the FND injection and imaged using the Maestro spectral imaging system. The dotted yellow circles indicate the location of the tumor. * Note: FNDs were located in the center of the tumor for five of the six mice. In the mouse treated anti-PD-L1 antibody and injected with dual-Ab FND, the FNDs were located adjacent to the tumor. (**b**,**c**) Tumors and spleens were harvested and processed into single-cell suspensions, and analyzed via flow cytometry. MDSC were characterized by CD11b^+^/GR1^+^ expression. The percentage of MDSC that were FND+ in the (**b**) tumor and (**c**) spleen was determined via fluorescence in the PE-Cy5.1 channel. Bars indicate mean ± SEM.

To evaluate the ability of FND to target MDSC in the setting of immunotherapy, FND were intra-tumorally injected into EMT6 tumor-bearing mice during the course of anti-PD-L1 therapy. Six EMT6 tumor-bearing BALB/c mice were treated with an anti-PD-L1 antibody, and six untreated mice served as controls. Twenty-four hours after the fourth dose, mice were given a 100 µL intra-tumoral injection of 1 µg/µL uFND, IgG FND, or dual-Ab FND. Animals were sacrificed 24 h after the FND injection and then imaged using the Maestro in vivo imaging system. In control mice, there was strong uptake in the tumor of dual-labeled FND and IgG-labeled FND as measured by fluorescence (Figure 6a). Administration of anti-PD-L1 therapy led to reduced uptake of the dual-labeled FND but not IgG-labeled FND (Figure 6a). Moreover, 24 h following intra-tumoral injection, FND tumors and spleens were harvested and processed into single-cell suspensions, and the percentage of FND+ MDSC was determined using flow cytometry (Figure S8). Approximately 10–20% of MDSC in the tumor were FND+ regardless of PD-L1 administration or type of FND injected. However, there was a trend toward reduced uptake of dual-Ab FND in mice treated with anti-PD-L1 compared to control (Figure 6b). Consistent with the results in Figure 4, there was low uptake of FND in spleens, with the highest percentage of splenic MDSC positive for FND being 1.5% from control mice treated with uFND (Figure 6c).

## 4. Discussion

Fluorescent nanodiamonds are unique nanoparticles that are chemically inert and demonstrate infinite photostability in the near-infrared region. FNDs exhibit minimal toxicity in vivo and have a modifiable surface chemistry, allowing for conjugation to various molecules and proteins. Due to these features, FNDs show promise as a therapeutic/theragnostic agent in medicine, especially cancer therapy. In the present study, we created fluorescent nanodiamonds that target murine MDSC via the conjugation of anti-CD11b and anti-GR1 antibodies to their surface. Successful antibody conjugation was demonstrated through ELISA capture and kinetic HRP experiments. Dual-Ab FND showed preferential uptake in vitro by a murine MDSC-like cell line, MSC2, as well as by purified murine MDSC from tumor-bearing mice. Dual-Ab FND showed significantly higher uptake by MDSC as compared to control IgG FND (94.7% vs. 69.0%, *p* < 0.05). Guided spectral imaging analysis of a murine model of hormone receptor-negative, HER2-negative (triple-negative) breast cancer showed fluorescent localization in the tumor at 2 and 24 h following intra-tumoral injection of FND, with less fluorescence noted in the spleen at both time points. Flow cytometric analysis of these samples confirmed that tumor and immune cells in the tumor had significantly greater uptake of dual-Ab FND than splenocytes. There was a trend toward a decrease in the percent of tumor and immune cells in the tumor positive for dual-Ab FND compared to uFND (7.6% ± 2.2 vs. 2.3% ± 1.1, *p* = 0.183). This decrease in total FND+ cells may be due to dual-Ab FND displaying reduced non-specific binding compared to uFND. Indeed, there was a trend toward increased specificity in the dual-Ab FND-treated tumors compared to uFND-treated tumors 2 h post-injection, indicated by an increase in the percentage of MDSC among all FND+ cells in the tumor (uFND: 77.2% ± 2.4 vs. dual-Ab FND: 87.1% ± 3.4, *p* = 0.259). However, an increase in specificity of the dual-Ab FND for MDSC was not observed at 24 h post-injection. Overall, the dual-Ab FND were quite specific to intra-tumoral MDSC, representing 87.1% and 83.0% of all FND+ cells 2 and 24 h post-injection, respectively. Furthermore, therapy with a PD-L1 blocking antibody had no significant effect on dual-Ab FND or IgG FND uptake by MDSC in vivo.

Our group previously demonstrated the successful conjugation of IgG to FND and subsequent targeted activation of innate immune cells, specifically natural killer cells and monocytes [23,38]. The present study reinforces these findings and demonstrates the first use of dual-antibody conjugation of FND to target a specific immune cell population, namely, immunosuppressive MDSC. The double antibody FND conjugate was created using a novel procedure to make bi-functionalized FND with both amine and propargyl groups on the FND surface. Both the concentration of carbonate buffer, pH 8.5, and the order of functionalization were important factors in the creation of the bi-functional FND.

The conjugation of two distinct antibodies to FND allows for more precise cellular targeting and could have major implications on directed therapies. Despite the novelty of this dual-Ab conjugated nanodiamond, the methods used for conjugation did not involve complex chemistry. Indeed, our group was able to replicate the methods previously described for the conjugation of human IgG to FND [38]. Glycidol-coated FNDs containing both amine and propargyl groups were reacted with N,N’-disuccinimidyl carbonate (DSC) to create NHS ester groups. Using these reactive groups, anti-CD11b antibodies were conjugated to the nanodiamond surface. A subsequent copper-catalyzed “Click” reaction allowed for additional conjugation of modified (PEGylated) GR1 antibodies. Confirmation of successful conjugation was performed using both FT-IR and ELISA. The question arises about the effect of FND conjugation on the availability/accessibility of the antigen-binding and Fc regions of the antibodies. The ELISA data suggests that the Ab-binding regions of the conjugated Abs were intact and functional. The impact of a second Ab on the activity of the primary Ab was not explored in the current analysis and was assumed to be minimal, given the uptake of dual-Ab FND by MDSC. However, this is an area of current investigation.

The concept of modifying the surface chemistry of FND for cell targeting and labeling has been explored previously. However, being able to successfully target, fluorescently label, and potentially activate/deactivate specific cells of interest is crucial to further developing nanodiamonds as a therapeutic application. As mentioned, Suarez-Kelley et al. from our group previously conjugated non-specific human IgG antibodies to FND for innate immune cell activation [38]. In that study, there was both a demonstration of uptake of IgG-conjugated FND and subsequent immune activation in human monocytes and natural killer cells. When FND were injected intra-tumorally in a murine model of breast cancer, IgG FND were visualized in the tumor at 24 h without localization of fluorescence to the liver or kidney. There was minimal uptake in the spleen at 24 h, similar to our findings in the present study. This result indicates decreased systemic uptake of Ab-targeted FND without affecting in vivo fluorescence or associated host viability. Other studies have attempted neoplastic cell targeting and labeling using more non-specific biocompatible molecules, such as vascular endothelial growth factor, folate, and viral envelope proteins [44,45,46]. Slegerova et al. developed FND conjugated to a cyclic arginylglycylaspartic acid (RGD) peptide, which binds to integrin αvβ3, a surface molecule highly expressed on solid tumors. When bound, the cyclic RGD peptide can block integrin function with multiple downstream effects (i.e., inhibition of angiogenesis). Their group demonstrated highly selective uptake of these conjugated FND when cultured in vitro with U 87 MG (human glioblastoma) cells, compared to FND alone or FND plus free cyclic RGD (non-conjugated) [47]. Notably, this study was not attempted in vivo. Torelli et al. synthesized FND conjugated to an engineered single-chain vascular endothelial growth factor (scVEGF). This conjugated FND targets VEGF receptors (R), which, like integrin αvβ3, are abundantly expressed in growing tumors. In vitro, FND-scVEGF demonstrated high affinity to human endothelial cells, which also have high levels of VEGFR expression, compared to human foreskin fibroblasts, which have low levels of VEGFR expression. In a murine 4T1 breast cancer model, FND-scVEGF demonstrated preferential accumulation in tumors compared to untargeted FND after tumor harvest and evaluation by epifluorescence microscopy. However, this group was unable to visualize FND via external sensors [45]. Additional groups used FND targeted to folate receptors concentrated on human cancer cells. This study showed that FND conjugated to folic acid demonstrated preferential uptake in HeLa cells in vivo, entering the cells via the caveolin-dependent endocytosis pathway [46]. The applications of FND modification and conjugation are broad. However, the present study suggests the potential for in vivo targeting and external localization of FND to a specific immune population via antibody conjugation. The immunosuppressive MDSC population is localized primarily to the tumor and spleen, and thus, targeting this population may have fewer effects outside the targeted tumor microenvironment than other FND-conjugated biomolecules.

This study demonstrates the first successful conjugation of two antibodies to the FND surface for specific immune cell targeting and labeling. However, limitations were encountered. First is the high uptake of uncoated FND within MDSC. This phenomenon was previously demonstrated in studies involving other phagocytic immune cell lines, that is, monocytes, and appeared not to affect cell viability or functionality [23,48]. When the nanodiamond surface was modified by the glycidol coat or non-specific IgG, there was decreased uptake within the target cell line but increased when coupled to MDSC-specific antibodies. We did not specifically investigate MDSC viability and activation markers after FND uptake. A second issue in the present study is the systemic uptake of FND in vivo. Based on the present results, FND were strongly retained within tumoral MDSC. The spleen has a relatively high concentration of MDSC compared to other tissues. There was little evidence of systemic absorption of the FND with localization to splenic MDSC, although imaging and cellular uptake past 24 h were not analyzed. Based on imaging, there was no evidence of fluorescence within the murine liver or kidney, though this was not evaluated at a cellular level. The lack of systemic absorption and the localization of the FND to tumor MDSC are likely facilitated by the direct, intra-tumoral injection. However, depending on tumor location, direct injection may be challenging or impossible to perform clinically. Therefore, for these cases, other approaches will be needed to achieve targeted delivery following systemic administration. An additional set of experiments using PD-L1 blockade did not significantly reduce the uptake of FND in either tumors or spleens in vivo. The use of PD-1/PD-L1 antibodies could make these observations more relevant to patients receiving these drugs. However, future studies evaluating the efficacy of using FND-based therapeutics in combination with immunotherapy will need to include larger sample sizes.

The potential uses of FND for both biomedical research and clinical application are broad. In this study, we described the techniques used to create a novel, dual-antibody (i.e., CD11b^+^ and GR1^+^)-conjugated fluorescent nanodiamond to target and label a specific innate immune cell population, immunosuppressive MDSC. The preferential uptake of dual-Ab FND compared to controls was demonstrated in vitro in MSC2 cells, a murine MDSC-like cell line, and purified MDSC from tumor-bearing mice. In vivo, dual-Ab FND were preferentially found in tumors compared to the spleen at both 2 and 24 h post-injection. Additionally, nanodiamonds were visualized via in vivo imaging at both time points. It was also demonstrated that the uptake of fluorescent nanodiamonds by tumor MDSC was not affected in mice treated with anti-PD-L1 immunotherapy. These findings could have implications for further studies looking at other phagocytic immune cell targets, such as tumor-associated macrophages or immunosuppressive dendritic cells. Additional studies investigating the conjugation of cytokines, chemotherapeutics, or other molecules to FND will hopefully enhance the current approach to targeted cancer therapies.

## Data Availability

The datasets used and analyzed during the current study are available from the corresponding author upon reasonable request.

## Supplementary Materials

The following supporting information can be downloaded at: https://www.mdpi.com/article/10.3390/nano14181509/s1, Figure S1. Mass spectra of synthesized and commercial GPE; Table S1. Flow cytometry antibodies; Figure S2. Effects of carbonate concentration on limited epoxidation of aminated FND; Figure S3. FT-IR results for the presence of propargyl-aminated FND; Figure S4. Flow cytometry gating strategy for Figure 4; Figure S5. Representative flow cytometry plots of the percentage of FND+ cells in the spleen following injection; Figure S6. Flow cytometry gating strategy for Figure 5; Figure S7. Representative flow cytometry plots of the percentage of MDSC among FND+ cells 24 h following injection; Figure S8. Flow cytometry gating strategy for Figure 6.
