# Supplementary material for: Targeting Myeloid-Derived Suppressor Cells via Dual-Antibody Fluorescent Nanodiamond Conjugate
Source: Nanomaterials (Basel). 2024 Sep 17;14(18):1509. doi: 10.3390/nano14181509 (PMC11434946; doi:10.3390/nano14181509)
Supplement: Supplementary file 1 [file nanomaterials-14-01509-s001.zip › nanomaterials-3156608-supplementary.docx]

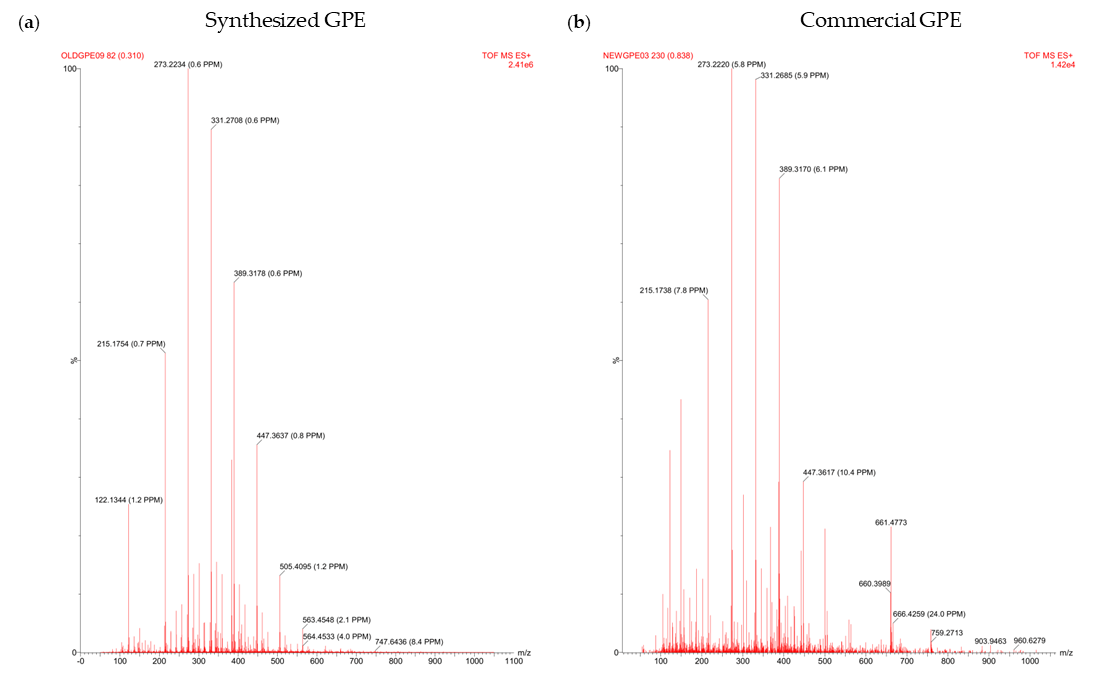


**Figure S1.** Mass spectra of synthesized and commercial GPE. (a) The mass spectrum of synthesized GPE has a series of m/z peaks at 215.175, 273.223, 331.271, 389.318, and 447.364. These peaks are all separated by 58.045 to 58.048 mass units, indicating a repeating polymer. (b) The mass spectrum of the commercial GPE has a similar pattern of major m/z peaks. The expected m/z peak of protonated GPE, 113.13, is not observed.**Table S1.** Flow cytometry antibodies.

| **Maker** | **Fluorophore** | **Manufacturer** | **Catalog no.** |
| --- | --- | --- | --- |
| CD45 | FITC | BioLegend | 103108 |
| CD11b | Brilliant Violet 421 | BioLegend | 101235 |
| CD11b | APC | BioLegend | 101212 |
| GR1 (Ly6-G/Ly6-C) | Alexa Fluor 488 | Invitrogen | RM3020 |
| GR1 (Ly6-G/Ly6-C) | Brilliant Violet 421 | BioLegend | 108433 |

**
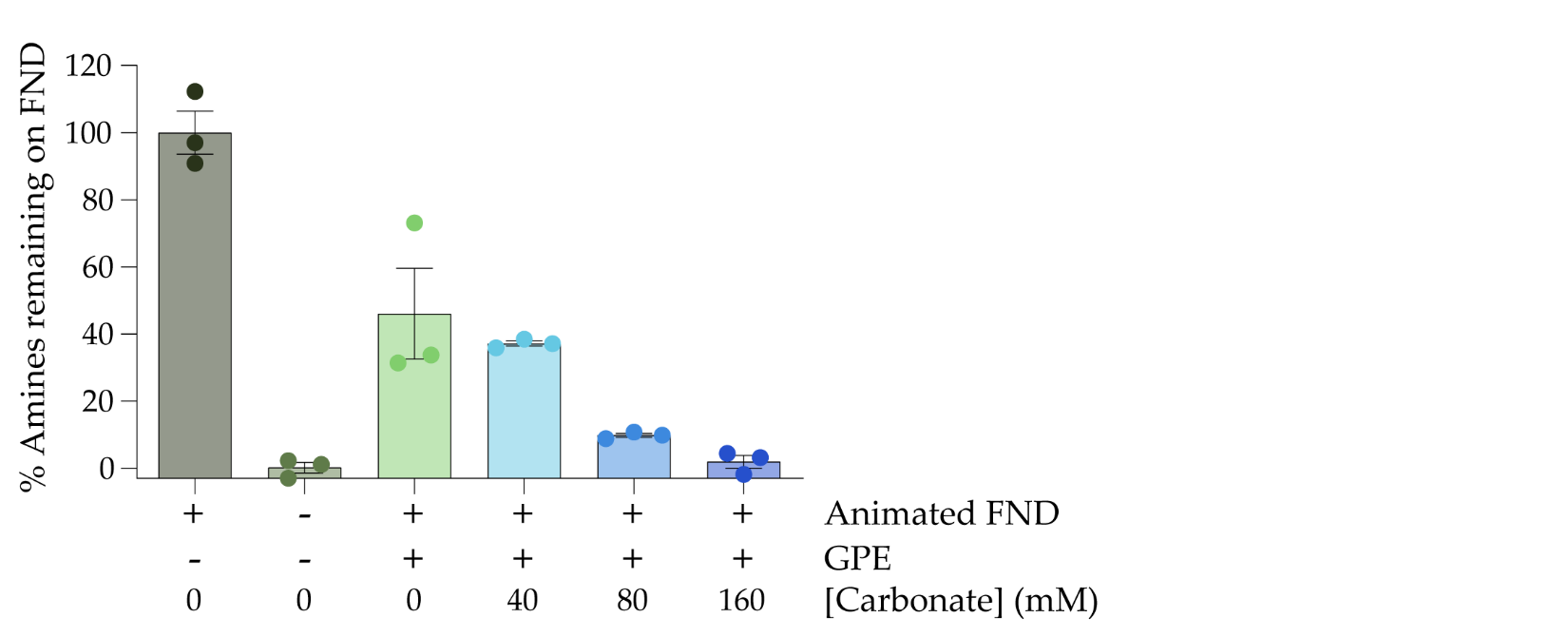
**

**Figure S2.** Effects of carbonate concentration on limited epoxidation of aminated FND. Aminated FND were reacted with GPE in a reaction buffer containing the indicated concentration of carbonate buffer (pH 8.5). After overnight epoxidation, the FND were reacted with SPDP, and the number of unreacted amines was estimated by the release of pyridine-2-thione. Results were normalized to aminated FND that were reacted with water. Non-aminated FND was used as a negative control. Bars indicate mean ± SEM.


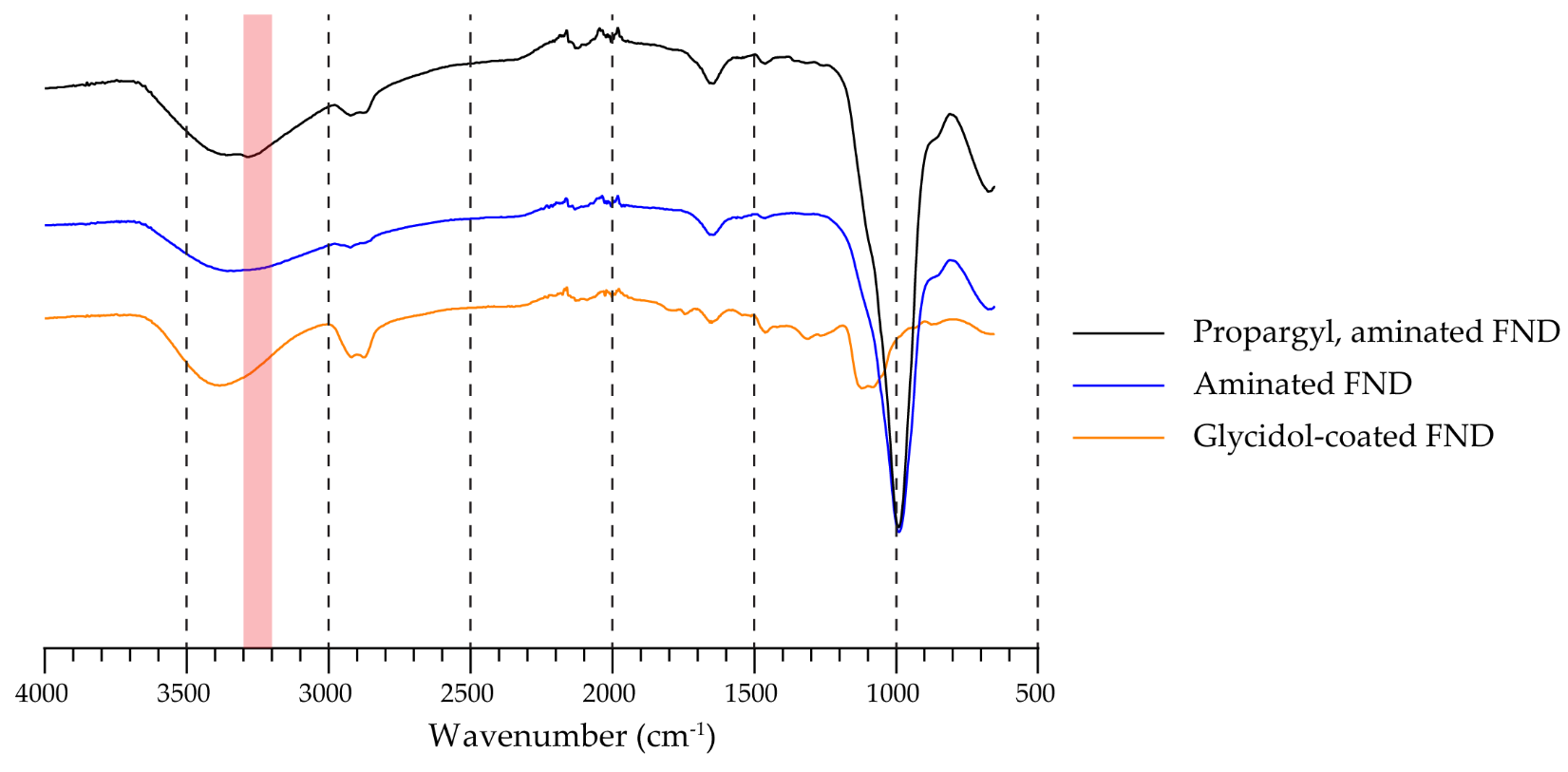


**Figure S3.** FT-IR results for the presence of propargyl-aminated FND. FT-IR results for FND indicate the presence of propargyl and amine groups on the FND surface (black line). The glycidol-coated FND (orange line) have a broad peak at 3400 cm^-1^ from the –OH groups as well as two peaks at 2870 and 2920 cm^-1^ from the CH_2_ stretching in the polymerized glycidol. These peaks are present in all three spectra. The amine diamonds have a larger peak at 1650 than the glycidol-coated FND, indicating the NH_2_ bending mode. When the amine diamonds are reacted with GPE (black line), a small peak at 3270 appears. This is attributed to the alkyne C-H stretching mode of the propargyl group. The peak at 1650 cm^-1^ is again larger than the same peak of the glycidol-coated FND, indicating there are amine groups that remain unreacted.

**
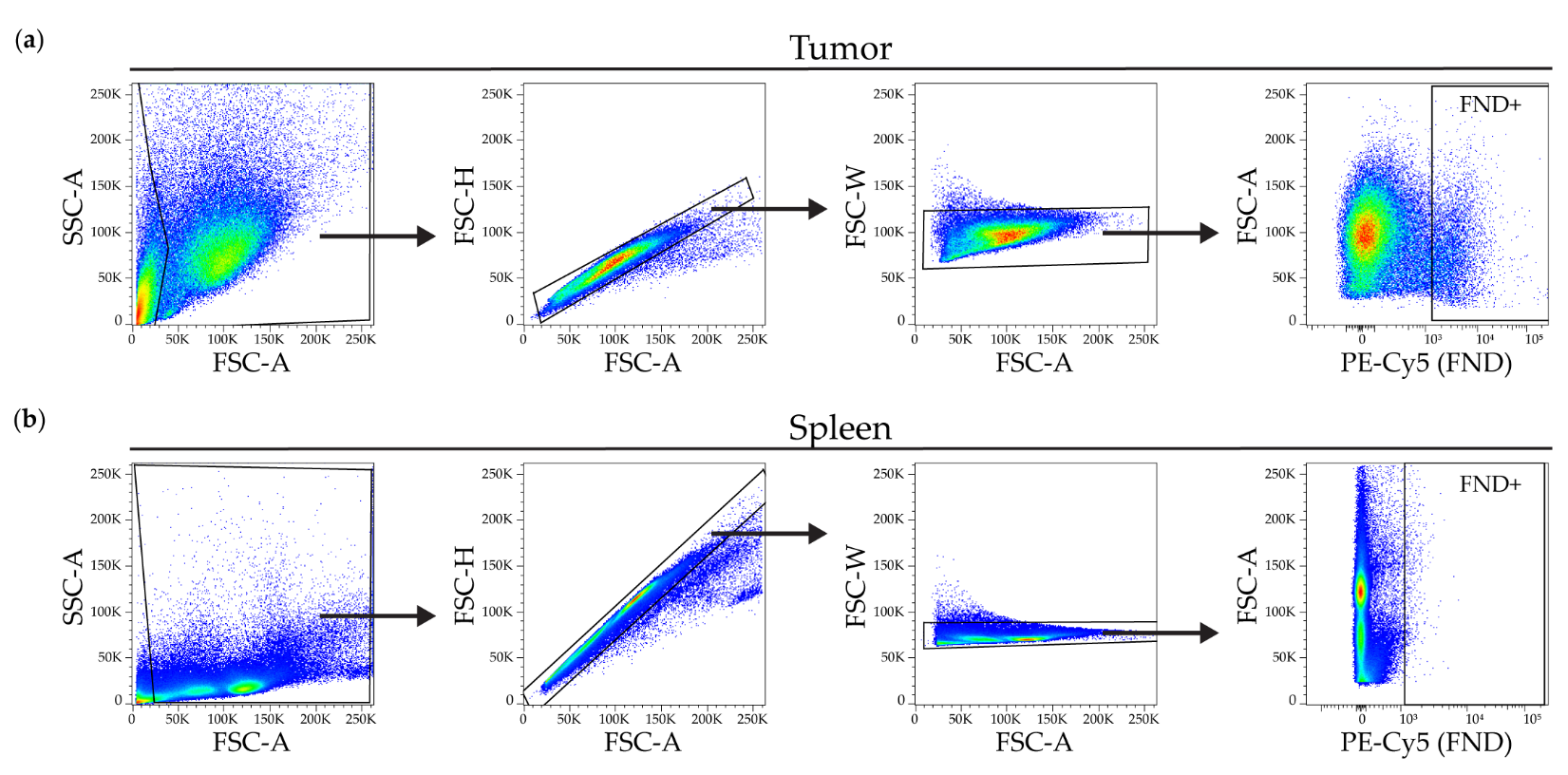
**

**Figure S4.** Flow cytometry gating strategy for Figure 4. Cells were gated on FSC-A vs. SSC-A, followed by FSC-A vs. FSC-H and FSC-A vs. FSC-W to remove doublets. FND+ cells were characterized by fluorescence in the PE-Cy5.1 channel. Representative flow plots of cells from (**a**) tumor and (**b**) spleen following an intra-tumoral injection of 100 µL of 1 µg/µL FND. FSC-A, forward scatter area; FSC-H, forward scatter height; FSC-W, forward scatter width; SSC-A, side scatter area.

**
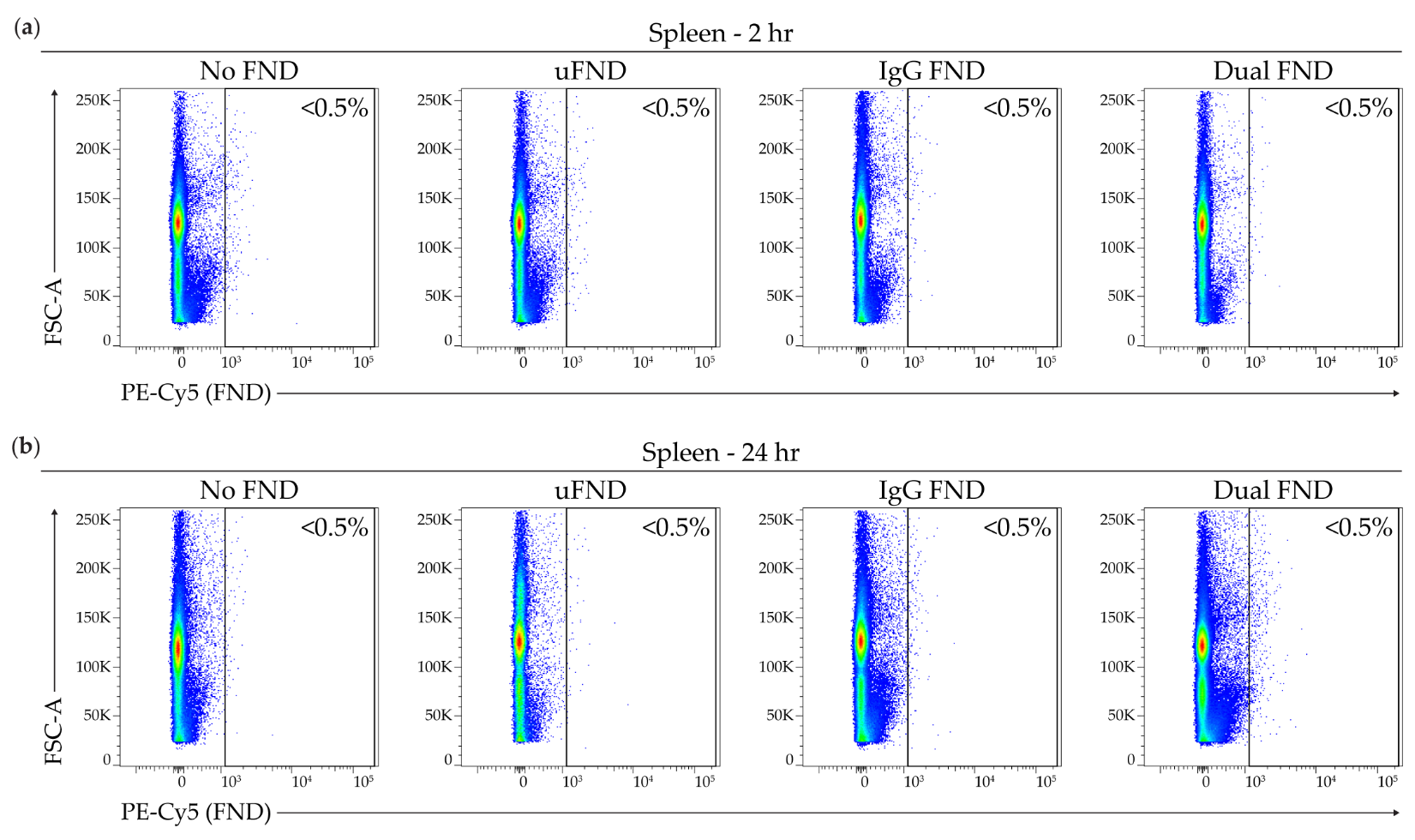
**

**Figure S5.** Representative flow cytometry plots of the percentage of FND+ cells in the spleen following injection. Mice bearing EMT6 tumors were treated as described in Figure 4. Representative flow cytometry plots demonstrating the percentage of FND+ cells in the spleen (**a**) 2 and (**b**) 24 hours following injection. FSC-A, forward scatter area.

**
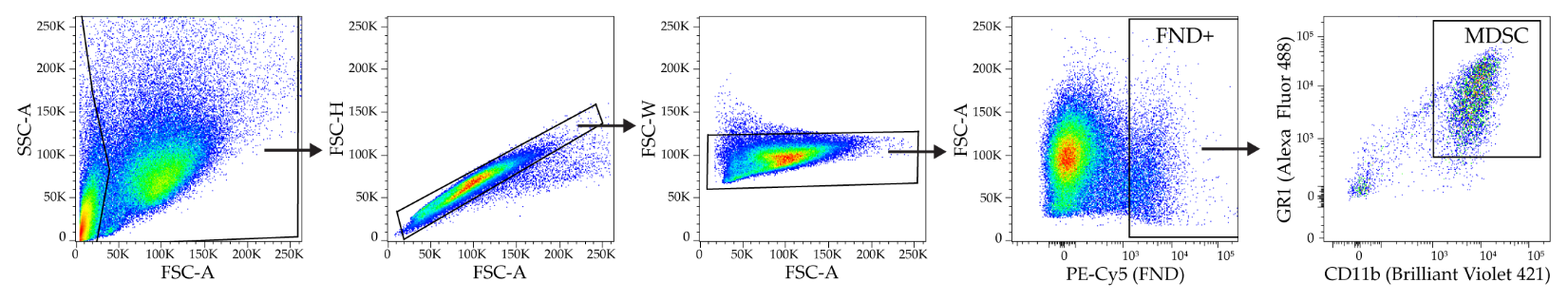
**

**Figure S6.** Flow cytometry gating strategy for Figure 5. Cells were gated on FSC-A vs. SSC-A, followed by FSC-A vs. FSC-H and FSC-A vs. FSC-W to remove doublets. FND+ cells were characterized by fluorescence in the PE-Cy5.1 channel, and MDSC were characterized by CD11b^+^/GR1^+^ expression. FSC-A, forward scatter area; FSC-H, forward scatter height; FSC-W, forward scatter width; SSC-A, side scatter area.

**
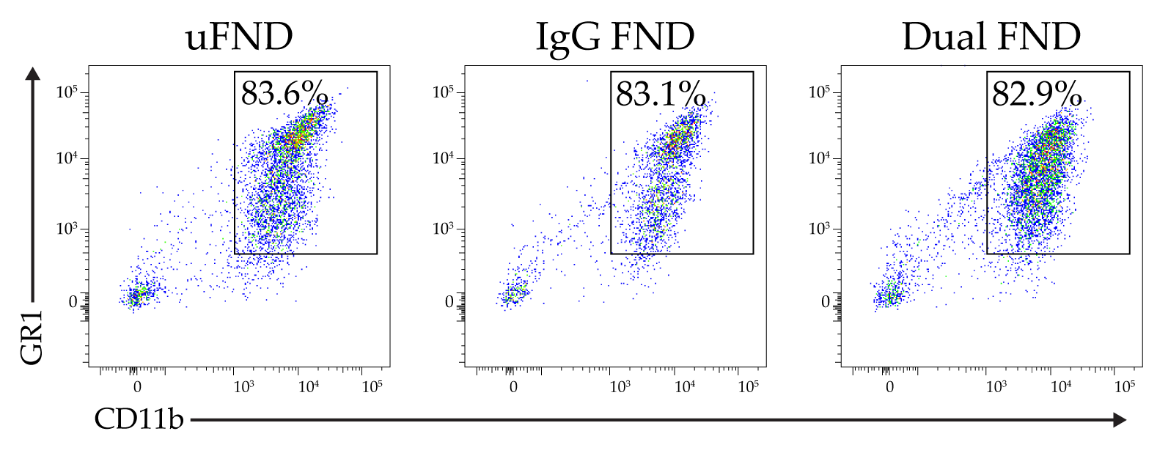
**

**Figure S7.** Representative flow cytometry plots of the percentage of MDSC among FND+ cells 24 hours following injection. Mice bearing EMT6 tumors were treated as described in Figure 5. Representative flow cytometry plots demonstrating the percentage of MDSC among FND+ cells in the tumor 24 hours following an intra-tumoral injection with 100 µL of 1 µg/µL (**a**) uFND, (**b**) IgG FND, and (**c**) dual-Ab FND. FSC-A, forward scatter area.

**
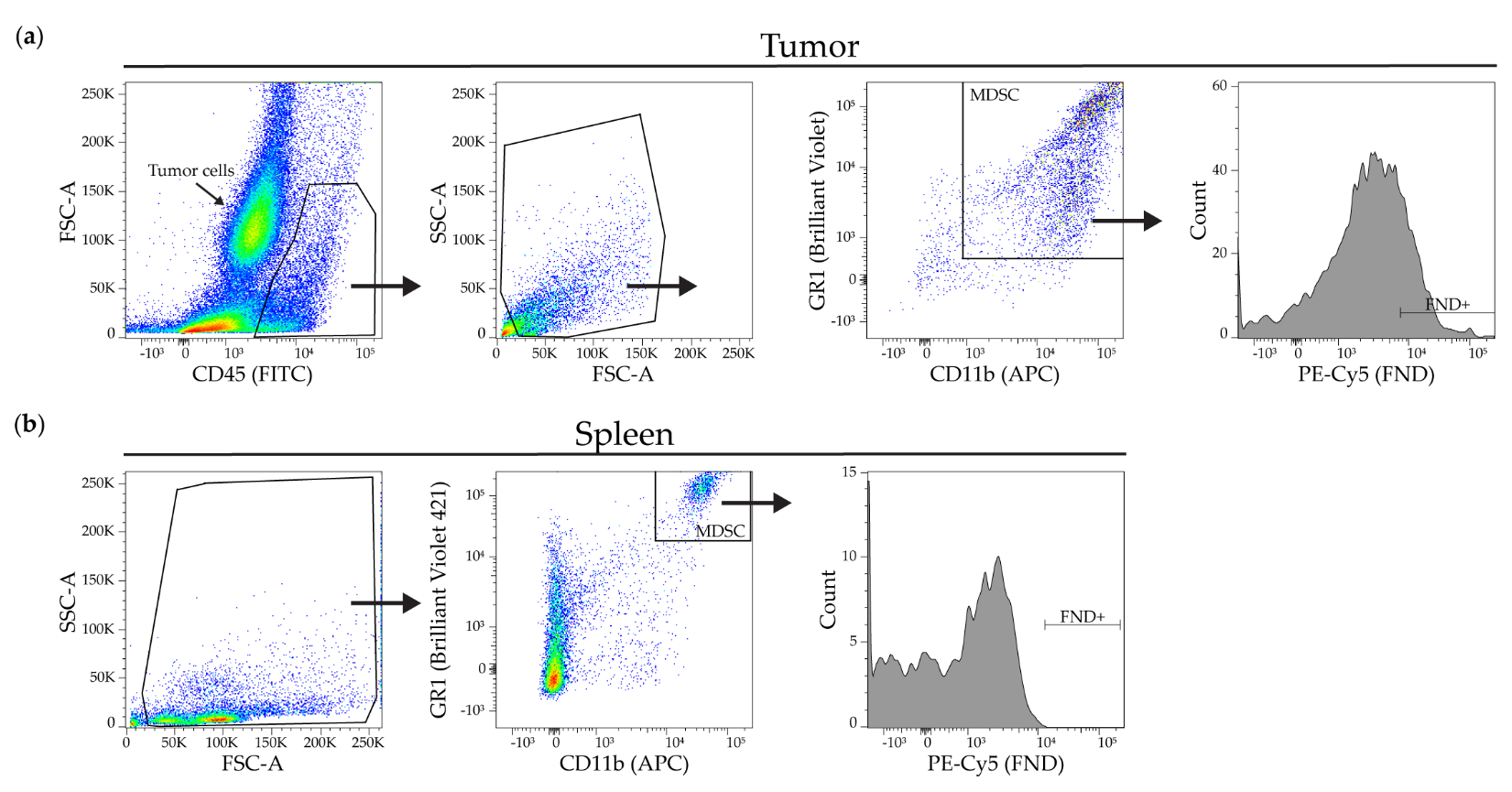
**

**Figure S8.** Flow cytometry gating strategy for Figure 6. (**a**) Cells from the tumor were first gated on CD45^+^ cells, followed by FSC-A vs. SSC-A. MDSC were characterized by CD11b^+^/GR1^+^ expression, and the percentage of FND+ MDSC was determined via fluorescence in the PE-Cy5.1 channel. (**b**) Cells from the spleen were gated on FSC-A vs. SSC-A, MDSC were by CD11b^+^/GR1^+^ expression, and the percentage of FND+ MDSC was determined via fluorescence in the PE-Cy5.1 channel. FSC-A, forward scatter area; SSC-A, side scatter area.
